# Associations of *Blautia* Genus With Early-Life Events and Later Phenotype in the NutriHS

**DOI:** 10.3389/fcimb.2022.838750

**Published:** 2022-05-12

**Authors:** Renata G. Borges de Oliveira Nascimento Freitas, Ana Carolina J. Vasques, Gabriel da Rocha Fernandes, Francieli B. Ribeiro, Isabela Solar, Marina G. Barbosa, Bianca de Almeida- Pititto, Bruno Geloneze, Sandra Roberta G. Ferreira

**Affiliations:** ^1^ Department of Epidemiology, School of Public Health, University of São Paulo, São Paulo, Brazil; ^2^ Laboratory of Investigation in Metabolism and Diabetes, Gastrocentro, School of Medical Sciences, University of Campinas, Campinas, Brazil; ^3^ School of Applied Sciences, University of Campinas, Campinas, Brazil; ^4^ Oswaldo Cruz Foundation, Belo Horizonte, Brazil; ^5^ Department of Preventive Medicine, Federal University of São Paulo, São Paulo, Brazil; ^6^ Obesity and Comorbidities Research Center, University of Campinas, Campinas, Brazil

**Keywords:** gut microbiota, early-life events, DOHAD, breastfeeding, nutritional status

## Abstract

**Introduction:**

Early-life events are associated with the risk of obesity and comorbidities later in life. The gut microbiota—whose composition is influenced by genetics and environmental factors—could be involved. Since the microbiota affects metabolism and fat storage, early-life insults could contribute to the occurrence of obesity driven, in part, by microbiota composition. We examined associations of gut bacteria with early-life events, nutritional status, and body composition in the Nutritionist’s Health Study (NutriHS).

**Methods:**

A cross-sectional study of 114 female participants examining early-life data, body composition, and biological samples was conducted. Fecal microbiota structure was determined targeting the V4 region of the 16S rRNA gene. Principal coordinates analysis (PCoA) and permutational multivariate analysis of variance (PERMANOVA) were used to test the impact of variables on microbial diversity. Profiles were identified using the Jensen-Shannon divergence matrix and Calinski–Harabasz index. Differential abundance between the categories of exclusive breastfeeding duration and nutritional status was tested using DESeq2.

**Results:**

In the sample [median age 28 years and body mass index (BMI) 24.5 kg/m^2^], 2 microbiota profiles driven by the *Blautia* or *Prevotella* genus were identified. An estimated 9.1% of the variation was explained by the profiles (p < 0.001), 2.1% by nutritional status (p = 0.004), and 1.8% by exclusive breastfeeding (p = 0.012). The proportion of participants with BMI <25 kg/m^2^ and who were breastfed for at least 6 months was higher in the *Blautia* profile (p < 0.05).

**Conclusion:**

Findings in a *Blautia*-driven profile of healthy women reinforce that early-life events play a role in defining gut microbiota composition, confirming the importance of exclusive breastfeeding for infant gut colonization in establishing a protective profile against adiposity-related outcomes in adulthood.

## Introduction

The importance of prenatal and postnatal events in long-term health outcomes has been consistently recognized (Ravelli et al., 1976; Barker, 1990; Bell et al., 2017; Block and El-Osta, 2017; Cheshmeh et al., 2020; Capra et al., 2021). Nutritional factors during intrauterine life and after birth have a major impact on infant health and later in adulthood, influencing the risk for non-communicable chronic diseases (Garmendia et al., 2014; Cadenas-Sanchez et al., 2017). Early feeding and infant growth rate have been associated with the risk of obesity and cardiometabolic diseases later in life (Kelishadi and Farajian, 2014; Kapourchali and Cresci, 2020). Important underlying mechanisms of these associations involve the gut microbiome (Bouter et al., 2017; Meijnikman et al., 2018). Gut colonization of the newborn starts at birth by bacteria from the mother and the environment. Major determinants of gut microbiota composition in early life are type of delivery, lactation, antibiotic use, and sanitary conditions (Biasucci et al., 2010; Martin et al., 2016; Le Doare et al., 2018; Cheng and Ning, 2019). Evidence indicates that these factors shape the gut microbiota throughout life (Rodríguez et al., 2015; Cheng and Ning, 2019) and that adult microbiota composition shows slight fluctuations around a core of stable colonizers.

Despite similar counts of human cells and microbes throughout the gastrointestinal tract, the gut microbiome contains 100 times more genes (Qin et al., 2010; Shen et al., 2013; Sender et al., 2016). This indicates that microbial communities play vital roles in the host and that an unbalanced microbiota can deteriorate regulatory functions, triggering immune and metabolic disturbances (Levy et al., 2017; Sommer et al., 2017). Factors such as aging (Rodríguez et al., 2015; Cheng and Ning, 2019; Fan and Pedersen, 2021), diet (David et al., 2014), nutritional status, and exercise induce changes (Rodríguez et al., 2015; Mailing et al., 2019) in microbiota composition, hampering understanding of the involvement of this complex ecosystem in pathophysiological processes. Arumugam et al. (2011) proposed analyzing the gut microbiota based on microbial profiles driven by discriminative genera referred to as enterotypes. Long-term dietary patterns have been linked to enterotypes in populations. A carbohydrate-based or vegetarian diet was found to be associated with *Prevotella*, while the typical Western diet was associated with *Bacteroides* enterotype (De Filippo et al., 2010; Wu et al., 2011; de Moraes et al., 2017). However, further studies have questioned such discrete profiles, given that these microbial communities proved not to be recurrent across diverse human populations (Gorvitovskaia et al., 2016). Despite controversies, it is clear that the risk or protection against non-communicable chronic diseases conferred by lifestyle is modulated by the gut microbiota, which affects nutrient acquisition, energy regulation, and fat storage (Rosenbaum et al., 2015; Wu et al., 2021). This could be a plausible pathway by which early-life exposures are associated with later body phenotypes.

Our group has been conducting the Nutritionist’s Health Study (NutriHS) involving nutrition undergraduates and nutritionists (Folchetti et al., 2016). This represents a unique opportunity to collect reliable nutrition-related data, accurate body composition measurements, and biological samples to test associations with early-life events and current lifestyle potentially mediated by the gut microbiota. The aim of the present study was to examine associations of gut bacteria with early-life events, current nutritional status, and body composition in NutriHS participants.

## Materials and Methods

### Study Design and Participants

This cross-sectional analysis was part of the multicenter NutriHS conducted at the School of Public Health of the University of São Paulo State, Brazil, to investigate markers of cardiometabolic diseases (Folchetti et al., 2016). Current data were collected at the University of Campinas (UNICAMP), located in Campinas city in the interior of São Paulo state. The NutriHS was approved by the local research ethics committee, and volunteers signed an electronic informed consent form available on the *e*-NutriHS system (www.fsp.usp.br/nutrihs). Recruitment of volunteers took place between 2018 and 2019.

Eligibility criteria were female undergraduates or nutritionists aged 19–44 years, body mass index (BMI) between 18.5 and 39.9 kg/m², and individuals whose mothers were alive. Pregnant and nursing women or individuals with diabetes, kidney, heart, and liver diseases, or other severe systemic diseases, in use of medications affecting glucose metabolism and/or body adiposity, or in use of probiotics or antibiotics in the last 3 months were excluded. Participants filled out online structured validated questionnaires. Respondents were then invited to schedule a face-to-face visit for physical examination and collection of biological samples. A total of 248 women answered the questionnaires, 127 met the inclusion criteria, and 114 concluded the full protocol (**Figure S1**).

### Early-Life and Current Data

Regarding information about early-life events, participants were advised to consult birth cards and seek assistance from their mothers. Maternal data collected were pre-pregnancy age, education levels (<11; ≥11 years) and BMI, and gestational diabetes, hypertension, or other complications (yes; no), parity (0; ≥1 pregnancy), tobacco, alcohol, and/or drug use (no; yes), and type of delivery (vaginal; C-section). Maternal gestational weight gain and participants’ birth weight were obtained as continuous variables. Continuous data on participant birth weight and duration of exclusive breastfeeding were further categorized into <2.5 kg, 2.5–4.0 kg, or ≥4.0 kg and into <6 months or ≥6 months, respectively.

Current data collected were skin color (white; non-white), age, family income (<6; ≥6 minimum wages), and engagement in leisure time physical activity (no; yes). Physical activity was assessed using the short version of the International Physical Activity Questionnaire (Craig et al., 2003) validated for use in Brazil (Matsudo et al., 2001). Dietary intake was estimated using a validated food frequency questionnaire for the adult population living in São Paulo, with the previous year as the time frame (Selem et al., 2014). The questionnaire comprised 101 food items, and food equivalents in the USDA National Nutrient Database for Standard Reference were employed (Haytowitz et al., 2019).

### Clinical and Body Composition Assessment

Body weight was obtained using a digital scale, and height was measured using a fixed stadiometer. BMI was calculated, and nutritional status was classified according to the WHO standards (WHO, 2015). Adequate nutritional status was defined as BMI >18.5 and <25 kg/m^2^. Waist circumference was measured at the midpoint between the last rib and iliac crest using an inelastic tape.

Body composition was assessed using dual-energy x-ray absorptiometry (DXA) (GE Lunar iDXA^®^ with EnCore software, Madison, WI, USA) by a trained researcher. Instrument quality control was checked routinely according to the manufacturer’s instructions. Parameters of interest were measurements of total fat and visceral fat mass and of total and appendicular lean mass.

### Biochemical Analyses

After a 12-h overnight fast, blood samples were collected for biochemical determinations. Glucose and lipid profile [total cholesterol, high-density lipoprotein (HDL) cholesterol, and triglycerides] were measured using the glucose oxidase and enzymatic colorimetric methods, respectively. Low-density lipoprotein (LDL) cholesterol was calculated by the Friedwald equation. Plasma insulin was obtained using an automated two-site chemiluminescent immunometric assay (Immulite 1000 System, Siemens Health Diagnostics, USA). Homeostasis model assessment of insulin resistance (HOMA-IR) was calculated (Matthews et al., 1985). High-sensitivity C-reactive protein was determined by nephelometry using a BN ProSpec System (Siemens, Marburg, Germany).

Plasma concentrations of short-chain fatty acids (SCFAs: acetate, propionate, and butyrate) were measured by gas chromatography (Wang et al., 2019). Briefly, ethanol, n-hexane, and an internal standard (caprylic acid) were added to serum. Samples were centrifuged and transferred to specific vials, and pH was adjusted to 4.0. A calibration curve with 0.015–0.1 mg/ml SCFA was used in the quantification. Chromatographic analyses were performed using a gas chromatograph-mass spectrometer (model Coupled QP2010 Plus; Shimadzu^®^, Kyoto, Japan) and a fused-silica capillary Stabilwax column (Restec Corporation, USA) with dimensions of 30 m × 0.25 mm internal diameter and coated with a 0.25-µm-thick layer of polyethylene glycol. Samples were injected at 250°C using a 25:1 split ratio for feces or splitless. High-grade pure helium was used as the carrier gas with a constant flow rate of 1.0 ml/min. Mass conditions were as follows: ionization voltage, 70 eV; ion source temperature, 200°C; full scan mode in the 35–500 mass range with 0.2 s/scan velocity. The butyrate columns did not appear, since the concentration of the acid was not detectable in the samples.

### Gut Microbiota Analysis

Fecal samples were refrigerated within 24 h after collection, and aliquots were stored at -80°C until analysis. According to the manufacturer’s instructions, DNA was extracted using the Maxwell^®^ 16 DNA purification kit and the protocol was carried out on the Maxwell^®^ 16 Instrument (Promega, Madison, WI, USA). We used the primers and workflow to generate the amplicon from the V4 region of the 16s rRNA gene according to Penington et al. (2018). The amplicon library produced was sequenced on the Illumina MiSeq platform (Illumina, San Diego, CA, USA), according to the manufacturer’s instructions.

The raw read files were processed in the R environment using the dada2 package [10.1038/nmeth.3869] (Ombrello, 2020). The forward and reverse sequences were trimmed to 150 bases. Reads containing more than two expected errors were removed. Errors in filtered sequences were corrected by the algorithm and joined to form the amplicon sequence variants (ASVs). The chimeric sequences were also removed, and a sample count table was generated. The taxonomic classification was done with the *tag.me* package [10.1101/263293] using the model 515F-806R (Pires et al., 2018).

### Statistical Analyses

All data were recorded, edited, and entered using the Statistical Package for the Social Sciences (SPSS version 20; IBM, NY, USA) and the R package for microbiota analyses. Level of significance was set at a p-value of 5%. Descriptive data were expressed as means [standard deviations (SDs)] or medians {q25–q75 ranges [interquartile range (IQR)]}. The Kolmogorov–Smirnov test was used to test data normality. Parametric tests (Pearson’s correlation coefficient and Student’s t test) and non-parametric tests (Spearman’s correlation coefficient and Mann–Whitney) were applied according to the distribution of variables.

The beta diversity was calculated using principal coordinates analysis (PCoA) and the ade4 R package for each library (Dray and Dufour, 2007). Permutational multivariate analysis of variance (PERMANOVA) was performed using 999 permutations to test the impact of categorical variables on beta diversity. Distance-based redundancy analysis (dbRDA) highlights variables with some association with the individual microbiota dissimilarities (Legendre and Anderson, 1999). Profiles were identified based on the Jensen-Shannon divergence matrix and using the Partitioning Around Medoids (PAM) algorithm, and the optimal number of clusters was determined by the Calinski–Harabasz index. The alpha diversity was measured by the Shannon and Simpson indexes. Differential abundance between profiles according to the categories of exclusive breastfeeding duration and nutritional status was tested using DESeq2, leaving genus with at least 50%-fold change and present in half of the samples (Love et al., 2014).

Macronutrient intakes were expressed as percentage of total energy intake (TEI) and fatty acid intake in grams. Correlations between dietary components and SCFA concentrations and body adiposity parameters were tested using Spearman’s coefficient.

## Results

The sample of 114 participants had a median age of 28 (IQR 24–31) years; 41.6% were undergraduates and 58.4% were nutritionists. Sixty-one percent of the participants engaged in moderate physical activity regularly, none was a professional athlete, and 51.0% had normal BMI. Regarding maternal characteristics, 35% had higher-level education and 90% were normal weight before pregnancy and had no clinical complications during the pregnancy. In the total sample, there was a predominance of cesarean delivery (66%) and normal birth weight (90%), 94% were breastfed, and 18% were exclusively breastfed for at least 6 months. In addition, 30% of participants reported overweight/obesity in childhood or adolescence.

**Figure 1:**
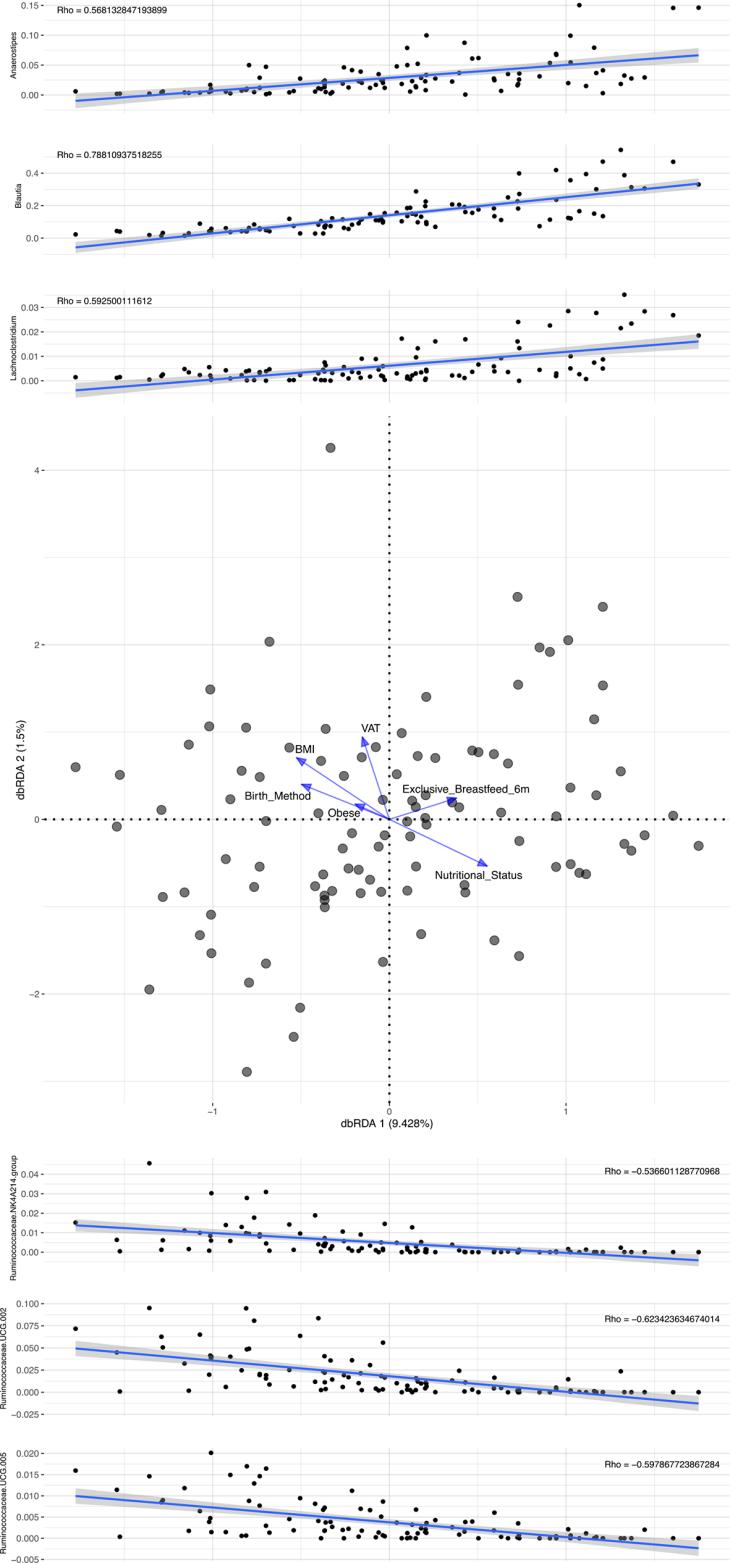
Distance-based redundancy analysis identified in 114 participants.

The dbRDA (**Figure 1**) results show that exclusive breastfeeding and adequate nutritional status are located to the right and adiposity parameters to the left of the plot. The relative abundance of *Blautia*, *Anaerostipes*, and *Lachnoclostridium* increased directly on the X-axis representing both breastfeeding and adequate nutritional status (**Figure 1**). Conversely, inverse relationships for *Ruminococcaceae* were observed. The results of the redundancy analysis for the most abundant bacteria are shown in the supplementary material (**Figure S2**).

**Figure 2:**
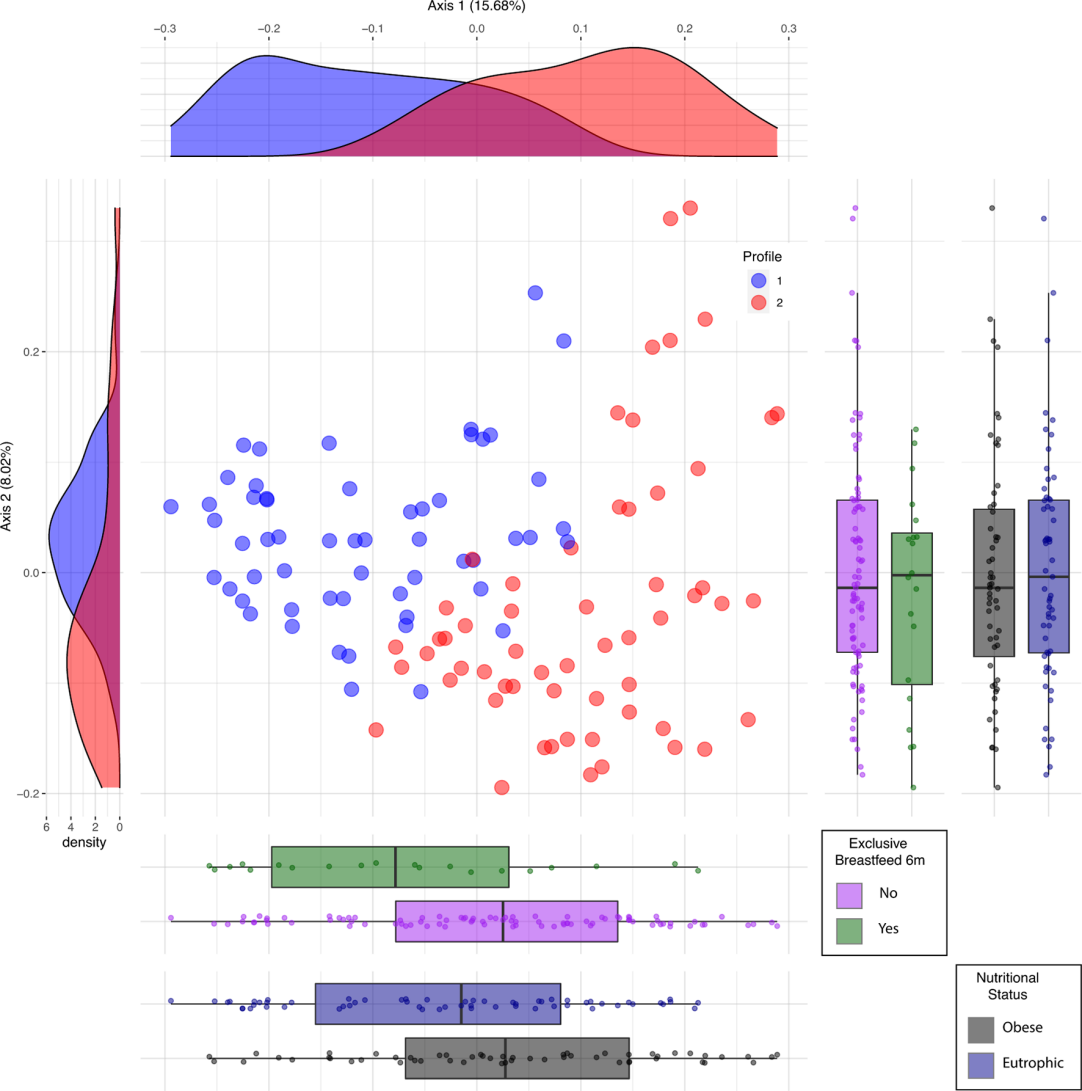
Profiles driven by *Blautia* (#1) and *Prevotella* (#2) identified by principal coordinates analysis (PCoA). #1 in blue is driven by *Blautia*; #2 in red is driven by *Prevotella*. Vertical boxplots represent the distribution of participants according to categories of breastfeeding and nutritional status (p < 0.05). Horizontal boxplots show the distribution of participants into profiles stratified by these categories.

**Figure 3:**
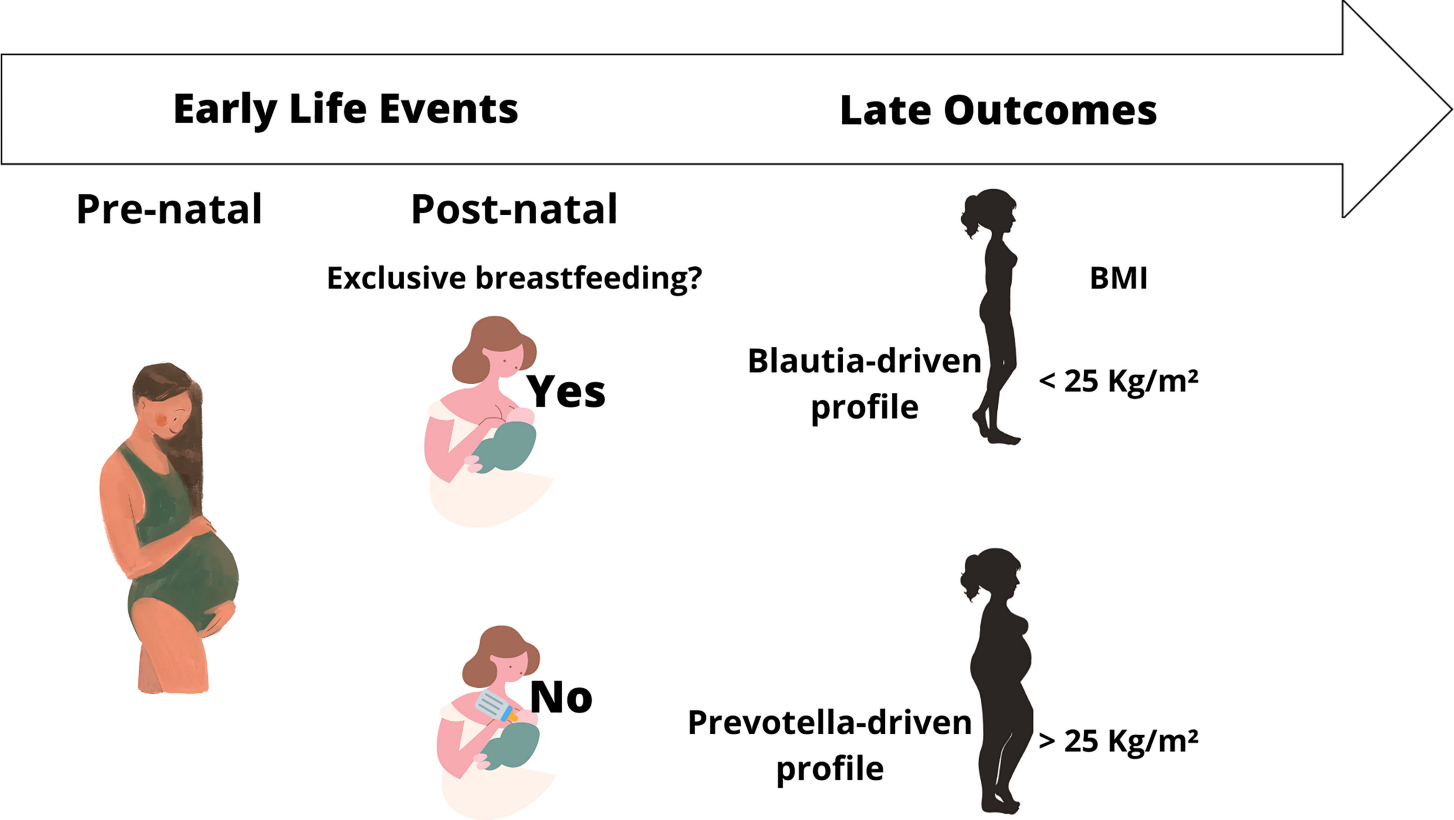
Impact of longer breastfeeding on microbiota composition and adult nutritional status. Credit: Figure produced using Canva graphic design platform (https://www.canva.com/) and brgfx/Freepik.

Beta diversity analysis of the microbiota revealed two bacterial profiles in the samples driven by the *Blautia* or *Prevotella* genus. Fifty-six participants were assigned to *Blautia* and 58 to *Prevotella* profiles. The PERMANOVA analysis on the Jensen-Shannon divergence values estimated that 9.1% of the variation among the samples was explained by the profiles (p < 0.001), 2.1% by nutritional status (p = 0.004), and 1.8% by exclusive breastfeeding (p = 0.012). Proportions of participants with BMI <25 kg/m^2^ and of those breastfed for at least 6 months were significantly (p < 0.05) higher in the *Blautia*-driven profile (**Figure 2**). A schematic interpretation of the main findings is provided in **Figure 3**. The proportion categorized by type of delivery (vaginal or cesarean section) or birth weight (adequate or inadequate) did not differ between the 2 groups (not shown in figures).

The differential abundance analysis identified genus drivers used to describe the bacterial composition in the profiles. The candidates present in at least 50% of the fecal samples are shown in the supplementary material (**Figure S3**), and bacteria differentially abundant between the 2 profiles were listed in **Table S1**.

**Figure 4:**
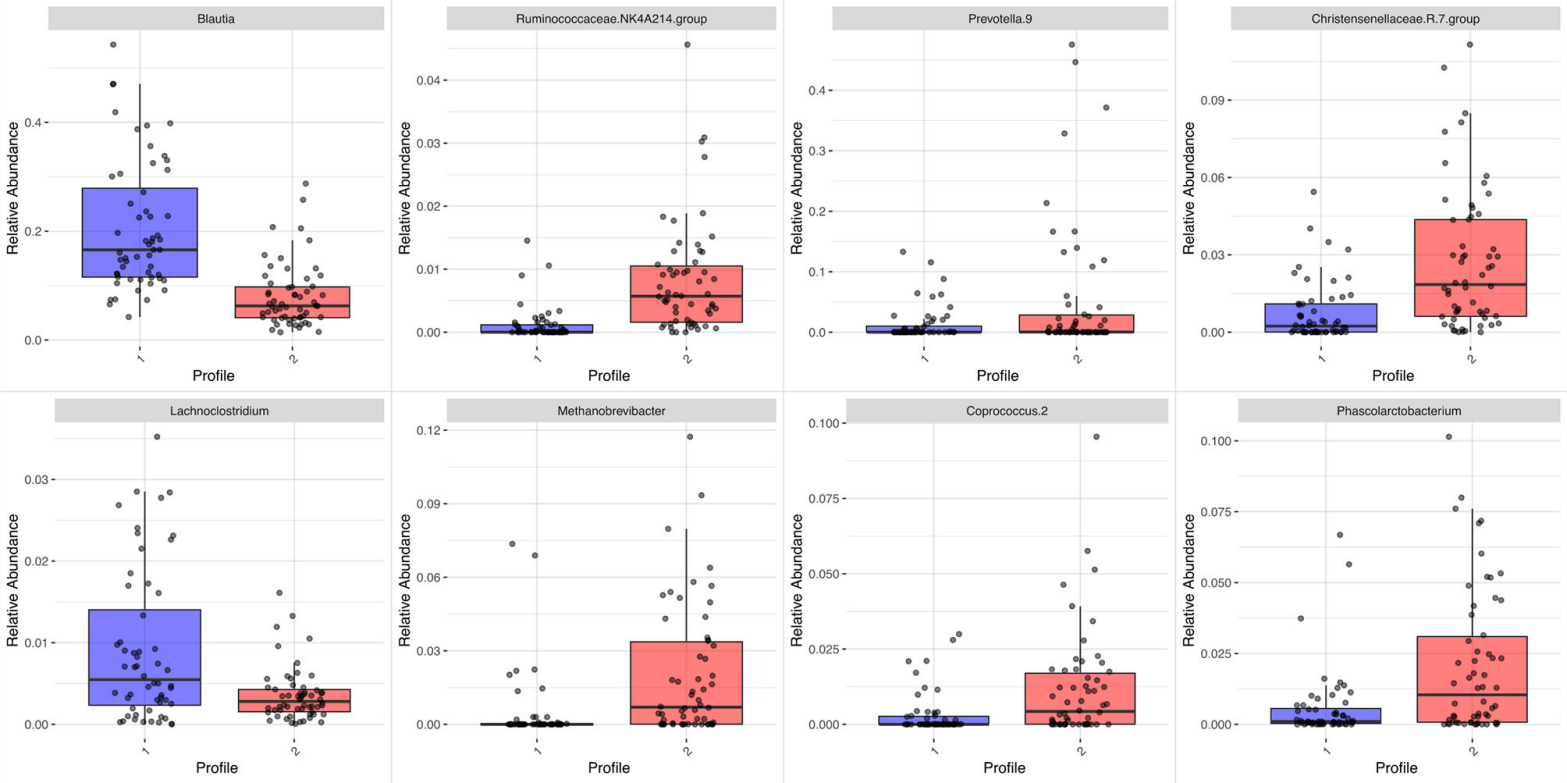
Boxplot of differential abundances of selected genera by profile (#1 in blue is driven by *Blautia*; #2 in red is driven by *Prevotella*). Adjusted p-value <0.05.

Differences in some abundances between the profiles are depicted in **Figure 4**. *Lachnoclostridium* (*Lachnospiraceae* family, *Clostridiales* order, *Clostridia* class) abundance was higher in the *Blautia* profile, whereas several genera from *Ruminococcaceae* and *Christensenellaceae* families (both from *Clostridiales* order, *Clostridia* class) were predominant in the *Prevotella* profile.

The main characteristics of participants by profile are given in **Table 1**. Gestational weight gain, type of delivery, and birth weight did not differ between the groups, but the rate of exclusive breastfeeding ≥6 months was higher in the *Blautia*- than that in the *Prevotella*-driven profile (21.4% vs. 6.9%, respectively, p = 0.04). Clinical and body composition variables of both groups were within normal ranges. Butyrate concentrations were undetectable for the whole sample.

**Table 1:** Means (standard deviation) or medians (interquartile range) for clinical variables and body composition parameters of the 114 participants according to profile.

	*Blautia* profile N = 56	*Prevotella* profile N = 58	p-value
• ***Early-life data***			
Pre-pregnancy maternal BMI (kg/m^2^)	21.8 ± 2.2	22.0 ± 2.8	0.77
Gestational weight gain (kg)	14.0 (9.0; 20.0)	12.0 (9.0; 16.0)	0.42
Type of delivery			0.89
- Normal, n (%)	36 (64.3)	36 (62.1)	
- Cesarean, n (%)	18 (32.1)	19 (32.8)	
Birth weight (kg)	3.2 ± 0.5	3.2 ± 0.4	0.92
Exclusive breastfeeding ≥6 months			0.04
- No, n (%)	39 (69.6)	43 (77.1)	
- Yes, n (%)	12 (21.4)	4 (6.9)	
*• **Clinical data***			
Body mass index (kg/m^2^)	23.9 (20.9; 28.1)	25.7 (21.7; 28)	0.25
Waist circumference (cm)	76.5 (71.1; 86.1)	79.1 (73.5; 91.0)	0.14
Fasting glucose (mg/dl)	82.9 ± 5.8	81.6 ± 5.8	0.26
HOMA-IR	1.2 (0.9; 1.7)	0.9 (0.7; 1.6)	0.12
HDL cholesterol (mg/dl)	58 (50; 67.5)	55 (49; 67)	0.29
Triglycerides (mg/dl)	79 (61; 103.5)	70 (59; 103)	0.45
C-reactive protein (mg/L)	1.2 (0.6; 2.7)	1.2 (0.6; 3.2)	0.59
Total short-chain fatty acids^a^ (mg/ml)	0.15 (0.10; 0.19)	0.13 (0.11; 0.20)	0.22
Acetate (mg/ml)	0.14 (0.09; 0.17)	0.11 (0.08; 0.14)	0.24
Propionate (mg/ml)	0.003 (0.002; 0.012)	0.004 (0.002; 0.011)	0.57
*• **DXA measurements***			
Total lean mass (kg)	38.1 ± 5.1	38.9 ± 5.1	0.44
Appendicular skeletal muscle mass (kg)	16.8 ± 2.8	17.2 ± 2.7	0.40
Total fat mass (%)	37.9 ± 6.6	38.5 ± 7.8	0.65
Android fat (%)	34.7 (29.4; 46.1)	35.2 (26.3; 47.4)	0.89
Gynoid fat (%)	43.1 ± 6.6	43.9 ± 7.4	0.54
Visceral adipose tissue (g)	141 (85; 435)	156 (87; 544)	0.49

Continuous variables were compared using Student’s t test or Mann–Whitney test, and data were expressed as mean ± standard deviation or median and q25–q75 ranges in parentheses. Categorical variables were compared using chi-square test.

a Total short-chain fatty acid = acetate + propionate.

HOMA-IR, Homeostasis model assessment of insulin resistance; HDL, high-density lipoprotein; DXA, dual-energy x-ray absorptiometry; BMI, Body mass index; HOMA-IR, Homeostasis model assessment of insulin resistance; HDL, high-density lipoprotein; DXA, dual-energy x-ray absorptiometry.

Dietary data of participants such as total energy and macronutrient and fatty acid intakes as a percentage of total energy did not differ, but median intakes of total, soluble, and insoluble fibers were higher in the *Prevotella*- than those in the *Blautia*-driven profile (**Table 2**). The dbRDA showed that the percentage of variance explained by diet was low (X-axis with 4.5% and Y-axis with 1.5%), being 94% explained by other factors. No significant correlation of fiber intake with SCFA concentrations and metabolic or body adiposity variables was detected.

**Table 2:** Medians (interquartile range) of total energy intake (TEI) and dietary data of the 114 participants according to profile.

	*Blautia* profile	*Prevotella* profile	p-value
**Total energy intake (kcal)**	1,958 (1,639; 2,223)	2,011 (1,593; 2,685)	0.51
**Carbohydrate (% TEI)**	47.0 (40.3; 52.2)	47.0 (41.2; 53.5)	0.43
**Protein (% TEI)**	16.0 (14.5; 18.9)	17.0 (13.9; 19.3)	0.97
**Total fat (% TEI)**	37.0 (33.0; 40.5)	36.0 (30.9; 39.3)	0.72
**SFA (g)**	27.7 (23.2; 35.2)	28.0 (20.7; 37.1)	0.78
**MUFA (g)**	25.3 (21.6; 32.2)	26.0 (19.5; 33.9)	0.87
**PUFA (g)**	17.0 (11.7; 21.6)	16.3 (11.3; 22.4)	0.86
**Total fiber (g)**	20.8 (16.1; 26.1)	23.7 (19.5; 34.3)	0.02
**Soluble fiber (g)**	5.6 (4.6; 7.5)	6.7 (5.0; 9.6)	0.04
**Insoluble fiber (g)**	15.3 (11.5; 19)	16.9 (14.1; 24.7)	0.02

Variables were compared using the Mann–Whitney test.

SFA, saturated fatty acid; MUFA, monounsaturated fatty acid; PUFA, polyunsaturated fatty acid.

## Discussion

This study explores the discussion regarding the influence of early-life events on gut microbiota composition in adulthood. A specific sample of women with literacy in nutrition was investigated. The associations suggested that longer breastfeeding impacts both microbiota composition and nutritional status in adulthood. By using a clustering approach to define microbiota profiles, in one profile driven by the genus *Blautia*, the same associations were confirmed. Both *Blautia*- and *Prevotella*-driven profiles are consistent with a healthy diet rich in fibers with an adequate macronutrient distribution and were therefore expected in the individuals studied. The findings in this homogeneous sample revealed the presence of macrostructures in the gut microbiota dominated by *Blautia* or *Prevotella*, SCFA-producing genera associated with beneficial metabolic effects. Interestingly, *Blautia* was associated with exclusive breastfeeding, whose relevance for gut colonization and body systems programming has been previously reported, as well as its health implications throughout the life span. Our findings not only reinforce the relevance of early feeding but also suggest an impact on gut colonization that persists into adulthood, contributing to a beneficial microbiota pattern. Furthering this knowledge could help in the prevention of chronic diseases.

For the overall sample, direct associations of some genera (*Blautia*, *Anaerostipes*, and *Lachnoclostridium*) of the family *Lachnospiraceae* with the recommended practice of long breastfeeding to prevent chronic diseases (including those related to excess body adiposity) were suggested. In fact, the profile analyses showed that the same genera were also associated with adequate nutritional status in adulthood. These findings contrast with previous reports of an association of *Lachnoclostridium* species with adiposity (Zhao L. et al., 2017; Sun et al., 2020; Nogal et al., 2021) but are in line with other studies in which *Anaerostipes* abundance was associated with a lower risk of type 2 diabetes (Yang et al., 2018). With regard to the family *Ruminococcaceae*, the present dbRDA initially suggested a relationship with unfavorable body adiposity distribution that was not confirmed when correlations to visceral adipose tissue (VAT) were tested. Although *Ruminococcus*, *Anaerostipes*, and *Blautia* produce SCFA (Vital et al., 2014; Koh et al., 2016), which have beneficial metabolic actions (Kasubuchi et al., 2015; Zhao et al., 2018; Müller et al., 2019), other controversial associations have been reported. In Mexican children, these genera were directly associated with obesity (Vazquez-Moreno et al., 2021). Inconsistencies have highlighted the need of improving knowledge about the intestinal bacteria assemblages of individuals from different geographical regions.

Using a clustering approach, early-life events and current characteristics of the sample were compared to verify associations suggesting underlying mechanisms of diseases. It is noteworthy that most participants engaged in physical activity regularly and consumed a fiber-rich diet, factors known to impact microbiota composition. These conditions have also been associated with anti-inflammatory status and favorable clinical profile (Hemmingsen et al., 2017; Nyberg et al., 2020). Normal mean values of C-reactive protein and insulin resistance index (HOMA-IR) indicated a low risk for metabolic disturbances in participants of both profiles. The findings of high abundances of *Blautia* and *Prevotella* were expected, since these genera belong to *Lachnospiraceae* and *Prevotellaceae* families that have the ability to degrade complex polysaccharides into SCFAs (Biddle et al., 2013; Eren et al., 2015). Measurements of SCFA in feces and blood have represented an indirect way of assessing the effect of fermentable carbohydrates’ intake. The higher the intake of these carbohydrates, the higher the SCFA concentration (So et al., 2018), but, in addition to the substrate availability, SCFA production is affected by intestinal transit time and microbiota composition (Macfarlane and Macfarlane, 2003). Characteristics of the microbiota of our participants should be contributing to improve the status of inflammation and insulin sensitivity, desirable for the prevention of cardiometabolic diseases (Kasubuchi et al., 2015; Zhao et al., 2018; Canfora et al., 2019). However, in the present study, no significant correlation was detected among fiber intake, SCFA concentrations, body adiposity, or metabolic variables. Additionally, the dbRDA showed a low percentage of variance explained by diet. The homogeneity and healthy characteristics of the sample as a whole may have precluded the detection of significant associations between these variables, as well as differences between participants from each profile.

Interestingly, comparisons of early-life events between the profiles showed that participants in the *Blautia* group had a higher rate of longer duration of exclusive breastfeeding. Considering the importance of gut colonization during this stage of life and given that these microorganisms coexist with the host throughout the life span (Milani et al., 2017), the association found might prove relevant. The first 1,000 days of life are considered a critical developmental window for programming systems and influencing the risk for long-term outcomes (Gluckman et al., 2005; Capra et al., 2021). In addition to the mode of delivery, growing evidence points to the role of early-life nutrition in shaping the offspring’s microbiota (Arrieta et al., 2014; Rodríguez, 2014). Bacteria are transferred through human milk and influence immune and metabolic homeostasis. Our results suggest that longer exposure to human milk might be associated with abundance of the *Blautia* genus. Despite limitations of linking distant factors with the gut microbiota of grown-up children and young adults, the hypothesis raised is feasible, considering the beneficial effects attributed to these bacteria. Breast milk composition is complex, containing nutrients, bacteria, and many other compounds. Oligosaccharides—present in human milk but not in most formula—serve as prebiotics, i.e., substrates for fermentation favoring the growth of beneficial bacteria such as the *Bifidobacterium* genus, which uses them to produce SCFA (Bridgman et al., 2017). The breastfed participants may have had their microbiota shaped to favor an abundance of certain commensal genera over others. In this respect, *Blautia* genus shares properties with *Bifidobacterium* in producing SCFAs and improving gut barrier functions. An interesting finding of our group previously suggested that breastfeeding duration could influence the offspring’s adherence to a prudent dietary pattern and metabolic parameters in adulthood (Eshriqui et al., 2019; Eshriqui et al., 2020). Another latent factor that could underlie the microbiota variability is the maternal and paternal BMI before conception (Eshriqui et al., 2021; Freitas et al., 2021), but, according to the PERMANOVA adjustments, there was no association between these maternal variables and the offspring’s microbiota structure.

A variety of exposures throughout life should have a role in modulating the microbiota of our participants. The current healthy lifestyle of individuals from the *Blautia*-driven profile may be contributing to an adequate BMI and normal biochemical profile. It is known that exercise-induced cardiometabolic benefits are in part gut microbiota-mediated (Chen et al., 2018), but there is also evidence on the associations of early-life events with obesity and related diseases (Ptashne, 2007; Garmendia et al., 2014; Cadenas-Sanchez et al., 2017). Lack of breastfeeding and exposure to formula were shown to increase the risk of obesity in infancy and adulthood (Dietz, 2001; Kelishadi and Farajian, 2014), with clear involvement of gut microbiota in this association. There was a predominance of participants with BMI <25 kg/m^2^ in the *Blautia* profile. This finding is congruent with evidence that butyrate (Berni Canani et al., 2016; Takahashi et al., 2016; Wang et al., 2018) and acetate produced by *Blautia* contribute to reduce obesity by regulating G-protein-coupled receptors (Kimura et al., 2013; Liu et al., 2021). In animals, weight gain prevention by SCFA supplementation (Lu et al., 2016) raises the possibility of a novel strategy for controlling human obesity. Our data are also in agreement with previous studies conducted in Spanish children (Benítez-Páez et al., 2020) and in Japanese adults (Ozato et al., 2019). A growing body of evidence indicates the potential on a deeper understanding of the “gut microbiota–host metabolism” interplay for managing prevalent diseases in different populations.

In some respects, the differential abundance analysis showed unexpected results. In the *Blautia*-driven profile, characterized by a higher proportion of lean individuals, *Methanobrevibacter* was less abundant. A previous study addressing this genus reported opposite results; however, the study in question involved an older sample of both sexes and had different purposes and methodological approaches (Schwiertz et al., 2010). Acetate-producing *Lachnoclostridium* was more abundant, in contrast with associations found for diet-induced obesity in animals (Zhao et al., 2017; Sun et al., 2020) and with VAT in female twins (Nogal et al., 2021). Some investigators have speculated that *Lachnoclostridium* could also be a Trimethylamine (TMA)-producing bacteria and, *via* the Trimethylamine N-oxide (TMAO) pathway, may increase the cardiometabolic risk (Schugar et al., 2017). In the *Prevotella*-driven profile, there was a higher abundance of acetate and butyrate-producing bacteria. The *Christensenellaceae* R7 group, *Ruminococcaceae* NK4A214, and *Phascolarctobacterium* have been associated with a favorable cardiometabolic profile. The *Christensenellaceae* R7 group was associated with less VAT and more lean mass in elderly people (Tavella et al., 2021), while the *Christensenellaceae* R7 group, *Ruminococcaceae* NK4A214, and *Phascolarctobacterium* were inversely correlated to glucose metabolism disturbance (Naderpoor et al., 2019; Chen et al., 2021). In our study, *Coprococcus_2* was more abundant in the *Prevotella*-driven than the *Blautia*-driven profile. A high abundance of this genus has been described in women with polycystic ovary syndrome (Zhou et al., 2020) and high lifetime cardiovascular disease risk (Kelly et al., 2016). Therefore, it can be concluded that both bacterial profiles identified in the gut microbiota of healthy Brazilian women may include both beneficial and harmful bacteria. Rather than investigating the role of isolated bacteria for risk prediction, a better strategic approach might be to prevent diseases by focusing on the microbial balance and interactions in the host, submitted to multiple exposures during the life course in different habitats.

This study has limitations related to the sample size due to strict inclusion criteria and composition. The sample comprised highly educated women with a healthy clinical profile, precluding generalizing our results to other samples with different characteristics. The sample homogeneity likely led to the detection of fewer differences between profiles, despite using an accurate technique for assessing body compartments. Our study was not designed to establish a causal relationship between exposure and long-term outcomes. Memory bias was also a concern. In order to minimize this type of error, the study included only participants whose mothers were alive, since the evidence shows that mothers are able to report the early life of their offspring with acceptable precision almost 30 years later (Chin et al., 2017). Another limitation was the lack of information regarding several risk factors such as antibiotic use and stressful conditions known to influence microbiota composition from birth to adulthood.

In conclusion, findings in a bacterial profile driven by *Blautia* present in healthy Brazilian women reinforce that early-life events play a role in defining gut microbiota profile. While acknowledging the need for investigations with appropriate design to further explore this hypothesis, we highlight the relevance of exclusive breastfeeding for gut colonization in early life to guide the establishment of a protective microbiota against adiposity-related outcomes throughout life.

## Data Availability Statement

The datasets presented in this study can be found in online repositories. The names of the repository/repositories and accession number(s) can be found below: https://www.ebi.ac.uk/ena, PRJEB49536.

## Ethics Statement

The studies involving human participants were reviewed and approved by Comitê de Ética em Pesquisa UNICAMP (CAAE 79775817.4.1001.5404). The patients/participants provided their written informed consent to participate in this study.

## Author Contributions

Contributed to conception and design: RB, AV, GR, BA-P, and SF. Contributed to acquisition, analysis, or interpretation: RB, AV, GR, FR, IS, MB, BG, and SF. Drafted the article: RB and SF. Critically revised the article: RB, AV, GR, BG, and SF. Gave final approval and agreed to be accountable for all aspects of work, ensuring integrity and accuracy: RB, AV, GR, FR, IS, MB, BA-P, BG, and SF.

## Funding

This work was supported by the Foundation for Research Support of the State of São Paulo–FAPESP (grant 2018/11433-9, 2018/11401-0).

## Conflict of Interest

The authors declare that the research was conducted in the absence of any commercial or financial relationships that could be construed as a potential conflict of interest.

## Publisher’s Note

All claims expressed in this article are solely those of the authors and do not necessarily represent those of their affiliated organizations, or those of the publisher, the editors and the reviewers. Any product that may be evaluated in this article, or claim that may be made by its manufacturer, is not guaranteed or endorsed by the publisher.

## Supplementary Material

Supplementary Figure 1 Infographic work of the overall methodology

Supplementary Figure 2 Redundancy analysis (RDA) highlighting profile drivers

Supplementary Figure 3 Boxplot of differential abundances of genera by profile (#1 in blue is driven by *Blautia*; #2 in red is driven by *Prevotella*). Adjusted p-value <0.05.

Supplementary Table 1 Differential abundances of bacteria by profile
