# Supplementary figures and images for: Associations of Blautia Genus With Early-Life Events and Later Phenotype in the NutriHS
Source: Front Cell Infect Microbiol. 2022 May 12;12:838750. doi: 10.3389/fcimb.2022.838750 (PMC9134825; doi:10.3389/fcimb.2022.838750)

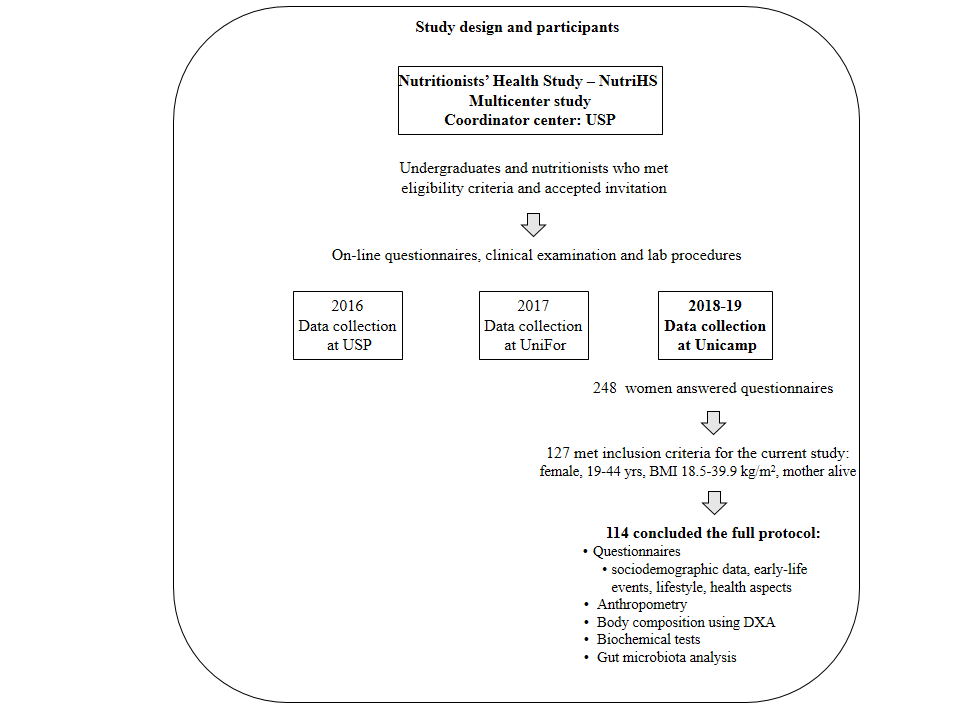

Supplement: Supplementary Figure 1 — Infographic work of the overall methodology [file Image_1.tif]

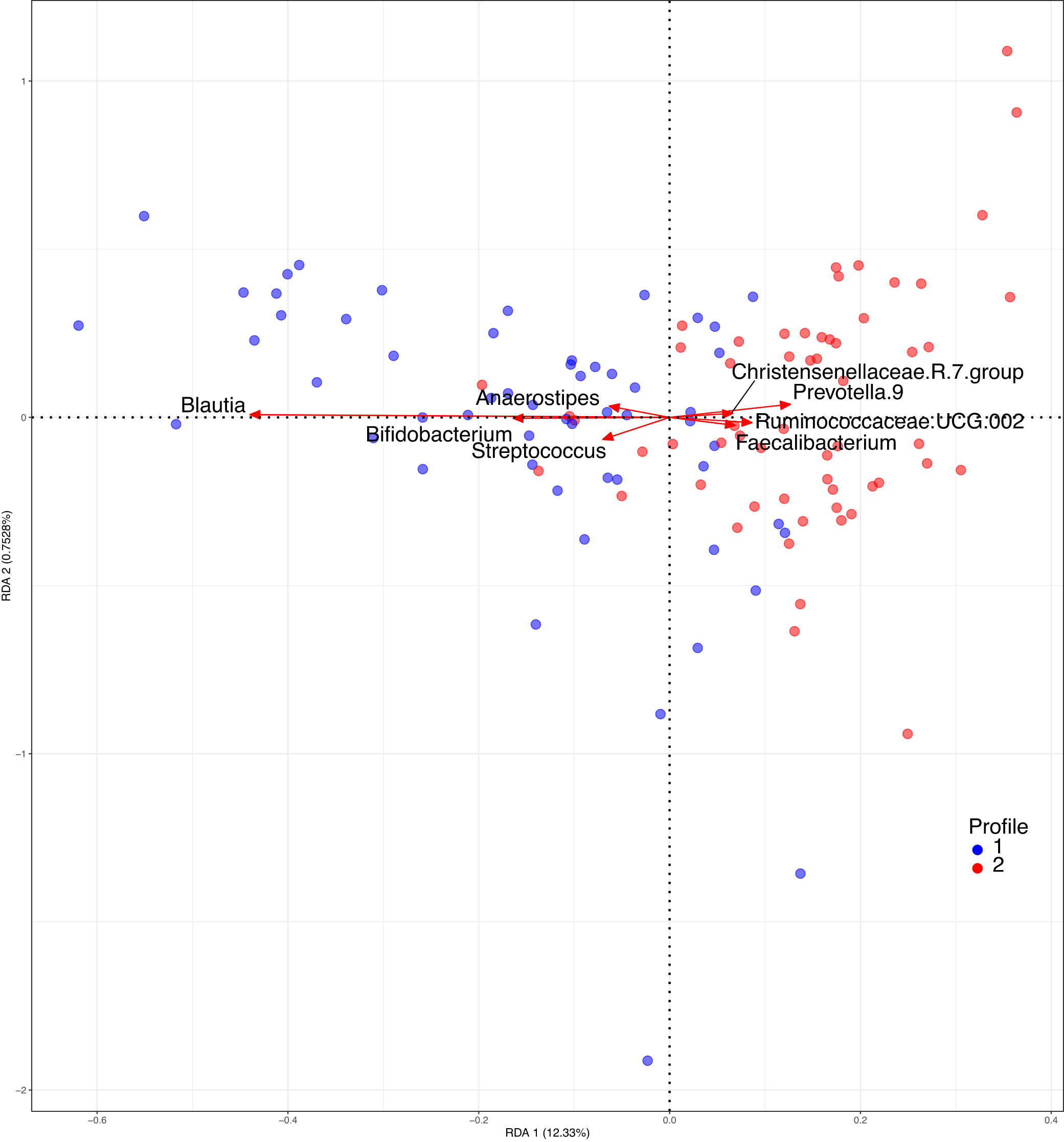

Supplement: Supplementary Figure 2 — Redundancy analysis (RDA) highlighting profile drivers [file Image_2.tiff]

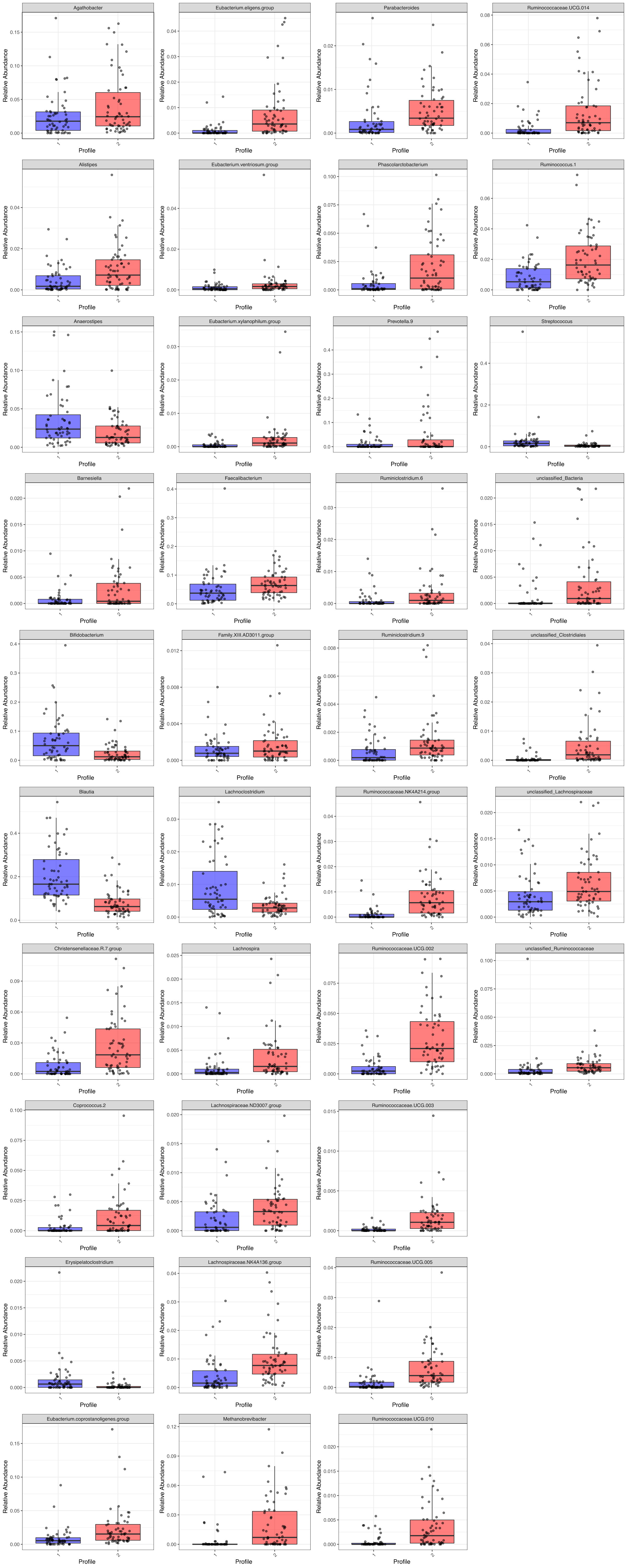

Supplement: Supplementary Figure 3 — Boxplot of differential abundances of genera by profile (#1 in blue is driven by Blautia; #2 in red is driven by Prevotella). Adjusted p-value <0.05. [file Image_3.tiff]
