# Supplementary material for: Associations of Blautia Genus With Early-Life Events and Later Phenotype in the NutriHS
Source: Front Cell Infect Microbiol. 2022 May 12;12:838750. doi: 10.3389/fcimb.2022.838750 (PMC9134825; doi:10.3389/fcimb.2022.838750)
Supplement: Supplementary Table 1 — Differential abundances of bacteria by profile [file Table_1.docx]

**Supplementary Material**

Bacteria that are differentially abundant in the two profiles, Blautia and Prevoltella, are listed in the Table 1S.

**Table 1S.** Differential abundances of bacteria in relation to profiles Blautia and Prevotella in participants of the NutriHS.

| Bacteria | baseMean | log2FoldChange | lfcSE | pvalue | padj |
| --- | --- | --- | --- | --- | --- |
| Agathobacter | 4577.23252425848 | 0.900045391390317 | 0.492370576612267 | 0.0014866264945607 | 0.00654919239495659 |
| Alistipes | 1043.25784355344 | 1.22030879757229 | 0.431552533848131 | 0.000147556443061386 | 0.00102809450572626 |
| Anaerostipes | 2631.09507779603 | -7.28479746407646e-07 | 0.00144266299212934 | 0.0098286302429425 | 0.0320413345919926 |
| Barnesiella | 242.217487154702 | 1.38891282415985 | 1.40894260320316 | 0.00323541831805848 | 0.0128627606303301 |
| Bifidobacterium | 6047.69869563105 | -1.33918903755783e-05 | 0.00144271813427867 | 0.0144326608951162 | 0.0435652541834062 |
| Blautia | 15803.6944688254 | -1.05810685692625 | 0.1672126653458 | 8.87273463129058e-12 | 4.82085248300122e-10 |
| Christensenellaceae R-7 group | 3086.96777310558 | 2.31801303047645 | 0.517123679611744 | 2.79439287336377e-07 | 4.55486038358294e-06 |
| Coprococcus 2 | 1119.47284622729 | 2.69408367629082e-06 | 0.00144269534740381 | 0.00425381044121023 | 0.0154082467092726 |
| Erysipelatoclostridium | 80.7587121659195 | -3.95318351126722e-06 | 0.00144269630762275 | 0.000607239007710205 | 0.00309312369552386 |
| Eubacterium coprostanoligenes group | 3107.4831887344 | 1.49592727997991 | 0.497605364938072 | 8.57725089940723e-05 | 0.000699045948301689 |
| Eubacterium eligens group | 789.924269497735 | 3.43862115335427 | 0.636693829427759 | 2.57246157751323e-09 | 8.38622474269312e-08 |
| Eubacterium ventriosum group | 224.718261636259 | 1.77145503295187 | 0.55777880297783 | 0.0103158354948395 | 0.0329702193266438 |
| Eubacterium xylanophilum group | 213.973823843391 | 2.75199352192944 | 0.693431201629131 | 2.43380450770802e-06 | 3.30591778963672e-05 |
| Faecalibacterium | 7988.36510473504 | 0.963563964481686 | 0.271011592673029 | 1.49159923931013e-05 | 0.000135072597781973 |
| Family XIII AD3011 group | 241.6963041379 | 4.41872783431115e-06 | 0.0014426917093278 | 0.00889690290890646 | 0.029595819880648 |
| Lachnoclostridium | 725.38887373369 | -0.904319173776392 | 0.325025534334357 | 0.000163990534655722 | 0.00102809450572626 |
| Lachnospira | 316.035475335843 | 2.04613717566843 | 0.553680098253514 | 7.49749863801952e-06 | 7.63807673748239e-05 |
| Lachnospiraceae ND3007 group | 461.975496333898 | 1.17046767181868 | 0.734885116013789 | 0.00198933505310594 | 0.00853320035937549 |
| Lachnospiraceae NK4A136 group | 1192.62598568395 | 1.64569901416262 | 0.443294445075076 | 7.48256340815602e-06 | 7.63807673748239e-05 |
| Methanobrevibacter | 2299.33621141309 | 1.66859194708965 | 1.7370092703479 | 0.00305941286895732 | 0.0124671074410011 |
| Parabacteroides | 484.929315870245 | 6.75889297252366e-06 | 0.00144269545403526 | 0.00471199314934418 | 0.0164480376050307 |
| Phascolarctobacterium | 2051.89973035165 | 1.96863936346024 | 0.580998221462612 | 2.37401758851682e-05 | 0.000203665719435917 |
| Prevotella 9 | 6990.871080842 | 2.24859281631551e-06 | 0.00144269528141787 | 0.00474268568979414 | 0.0164480376050307 |
| Ruminiclostridium 6 | 435.63137160963 | 2.42638212024755 | 0.886750681801896 | 0.000161836085309354 | 0.00102809450572626 |
| Ruminiclostridium 9 | 162.286880662844 | 1.31215073303986 | 0.639675116137761 | 0.000913216478879353 | 0.00437806723698043 |
| Ruminococcaceae NK4A214 group | 881.363327806308 | 3.05168436696597 | 0.631049198675696 | 4.95271604616085e-08 | 8.9699190613802e-07 |
| Ruminococcaceae UCG-002 | 3278.61940257742 | 2.61657034491116 | 0.529987401693509 | 3.14119865832892e-08 | 6.40019226634518e-07 |
| Ruminococcaceae UCG-003 | 153.226867075064 | 3.64085329250354 | 0.6608143888039 | 1.28209359722525e-09 | 5.22453140869288e-08 |
| Ruminococcaceae UCG-005 | 789.479920177445 | 1.80641012908677 | 0.616509086197927 | 0.000101856210346434 | 0.000768065525832578 |
| Ruminococcaceae UCG-010 | 444.284111329049 | 3.24594144421966 | 0.754316271216326 | 5.83643866565205e-07 | 8.64854093182985e-06 |
| Ruminococcaceae UCG-014 | 1601.320452623 | 2.74961455959421 | 0.715692642544521 | 4.17834064843921e-06 | 5.23899635150454e-05 |
| Ruminococcus 1 | 1930.92453056734 | 1.54542401532409 | 0.419413998434368 | 8.16081224238033e-06 | 7.82477879710585e-05 |
| Streptococcus | 1499.71837870942 | -2.39540270669191 | 0.285407565823045 | 8.57126859902823e-09 | 1.99588111663086e-07 |
| unclassified_Bacteria | 507.066001602359 | 2.7479111189258e-06 | 0.00144269533814265 | 0.00409408823178107 | 0.0154082467092726 |
| unclassified_Clostridiales | 554.962580449249 | 3.55691197696908 | 0.674927234913494 | 5.16438843583779e-09 | 1.40299219173593e-07 |
| unclassified_Lachnospiraceae | 766.33271952789 | 0.919844745236887 | 0.311242426949579 | 0.000103665285695195 | 0.000768065525832578 |
| unclassified_Ruminococcaceae | 1000.0777153551 | 1.13750671771947 | 0.469283918275103 | 0.000420494434472704 | 0.00228468642730169 |

^a^ FoldChange calculated as Log2 (Profile Prevotella Abundance/Profile Blautia Abundance)

Tested using DESeq2

Adjusted p-value < 0.05
